# Low levels of AMPK promote epithelial‐mesenchymal transition in lung cancer primarily through HDAC4‐ and HDAC5‐mediated metabolic reprogramming

**DOI:** 10.1111/jcmm.15410

**Published:** 2020-06-09

**Authors:** Shoujie Feng, Li Zhang, Xiucheng Liu, Guangbin Li, Biao Zhang, Ziwen Wang, Hao Zhang, Haitao Ma

**Affiliations:** ^1^ Department of Thoracic Surgery The First Affiliated Hospital of Soochow University Suzhou China; ^2^ Department of Thoracic Surgery Affiliated Hospital of Xuzhou Medical University Xuzhou China; ^3^ Thoracic Surgery Laboratory Xuzhou Medical University Xuzhou China; ^4^ Editorial Office of International Journal of Anesthesiology and Resuscitation Xuzhou Medical University Xuzhou China; ^5^ Intensive Care Unit The Affiliated Hospital of Xuzhou Medical University Xuzhou China

**Keywords:** AMPK, epithelial‐mesenchymal transition, lung cancer, metabolism

## Abstract

AMP‐activated protein kinase (AMPK) serves as a “supermetabolic regulator” that helps maintain cellular energy homeostasis. However, the role of AMPK in glucose metabolism reprogramming in lung cancer remains unclear. Here, our study shows that low AMPK expression correlates with metastasis and clinicopathologic parameters of non–small‐cell lung cancer. Low AMPK significantly enhances the Warburg effect in HBE and A549 cells, which in turn induces the expression of mesenchymal markers and enhances their invasion and migration. At the mechanistic level, low AMPK up‐regulates HK2 expression and glycolysis levels through HDAC4 and HDAC5. Collectively, our findings demonstrate that low AMPK‐induced metabolism can promote epithelial‐mesenchymal transition progression in normal bronchial epithelial cells and lung cancer cells, and increase the risk for tumour metastasis.

## 1 INTRODUCTION

Lung cancer has become a leading cause of cancer‐related deaths in China and worldwide. Deaths of most lung cancer patients are metastasis‐related.^1^ In recent years, with the advancement of radical surgical techniques, the advent of new molecular targeted drugs and the development of neoadjuvant therapy, prognosis of lung cancer patients has significantly improved.^2^ However, 5‐year overall survival in patients with lung cancer is still unsatisfactory. Therefore, seeking new ways to inhibit lung cancer metastasis is a common objective of thoracic experts.

In addition to tumour cells’ infinite proliferative capacity, loss of contact inhibition and migration ability, metabolic rearrangement is yet another important feature of malignant tumours.^3^, ^4^ To facilitate proliferation, invasion and escape from the immune system, tumour cells actively choose glycolysis as the main way to obtain energy, even in an oxygen‐rich environment, namely the Warburg effect. Due to the heterogeneity of cancer, there are significant differences in the metabolic profiles of different types of tumours, and tumours at different stages of development, further complicating things.^5^, ^6^

Epithelial‐mesenchymal transition (EMT) is a process in which the expression of epithelial markers, such as E‐cadherin decreases and the expression of interstitial markers (eg, N‐cadherin and Fibronectin) increases.^7^, ^8^, ^9^ For non–small‐cell lung cancer (NSCLC), it is ubiquitous for tumour cells to acquire increased mobility, invasiveness and apoptosis resistance through pathological EMT.^10^, ^11^ In some sense, EMT is also an adaptive behaviour of tumour cells, exchanging cellular demands for rapid proliferation for survival and metastasis. It is therefore plausible to assume that the EMT process and metabolic reprogramming of tumour cells are closely related. Studies have shown increased glucose uptake and metabolic enzyme expression in EMT‐transformed breast, lung and pancreatic cancer cells. However, the metabolic control points, the specific relationship between metabolic reprogramming and EMT in NSCLC cells, and related mechanisms still remain poorly defined. AMP‐activated protein kinase (AMPK) serves as a “supermetabolic regulator” that helps maintain cellular energy homeostasis.^12^ It is a heterotrimeric protein, consisting of a catalytic α‐subunit (isoforms α1 or α2), a regulatory β‐subunit (isoforms β1 or β2) and a nucleotide binding γ‐subunit (isoforms γ1, γ2 or γ3). The α‐subunit has a conventional serine/threonine kinase domain at the N‐terminus, with a conserved threonine residue.^13^ AMPK has been associated with several physiological processes including cell division, endothelial cell migration and maintenance of epithelial cells polarity. From a metabolic perspective, AMPK promotes ATP conservation, under conditions of metabolic stress. It activates catabolic metabolism pathways, such as autophagy, and inhibits anabolic processes.^14^, ^15^ A recent report by Jones et al^16^ showed that AMPK is a negative regulator of the Warburg effect and that it suppresses tumour growth. Despite evidence linking AMPK with tumour suppressor functions, the role of AMPK in tumorigenesis and tumour metabolism is still not known.

In this study, we specifically sought to address the following aspects: (1) what is the involvement of AMPK in tumorigenesis and progression of NSCLC; and (2) what are the mechanisms associated with AMPK’s effects on tumour metabolism and tumour metastasis.

## 2 MATERIALS AND METHODS

All experiments were performed in adherence with the National Institutes of Health (NIH Publication, 8th Edition, 2011) guidelines on the use of laboratory animals. The animal care and experimental protocols were approved by the Suzhou University Committee on Animal Care.

A series of human NSCLC specimens were obtained from the pathology department of affiliated hospital of Suzhou University. The patients’ clinical information includes age, sex, tumour differentiation, lymph node metastasis and TNM stage. All specimens were collected under the guidance of the HIPAA protocol and supervised by the ethics committee of the hospital.

### 2.1 Construction of tissues microarrays and immunohistochemistry

Tissues microarrays (TMA) were constructed by the Department of Pathology, the First Affiliated Hospital. For immunohistochemistry (IHC), after antigen retrieval using EDTA, the specimens were blocked with goat serum for 20 minutes before applying the primary antibody. Specimens were incubated with anti‐AMPKα (Abcam, cat #ab32047; 1:200) for 12 hours in 4°C. Next, the sections were washed twice and subsequently incubated with HRP‐polymer‐conjugated secondary antibody (Zhong Shan, China) at room temperature. Finally, the microarrays were then stained with 3, 3‐diaminobenzidine solution and haematoxylin. The slides were photographed with an inverted microscope (Olympus).

Two pathologists assessed separately the sections under blinded experimental conditions, and all differences that arise were resolved by discussion. The staining scores of AMPK were evaluated via combining the percentage of cells with the staining intensity and being dependent on the IRS. The intensity of AMPK immunostaining was scored as 0‐3 (0, negative; 1, weak; 2, moderate; 3, strong); the percentage of immunoreactivity cells was graded as 1 (0%‐25%), 2 (26%‐50%), 3 (51%‐75%) and 4 (76%‐100%). Relied on the IRS, the level of AMPK expression was categorized as low (IRS: 0‐6) and high (IRS: 8‐12) expression.

### 2.2 Animal feeding and treatment

BALB/c nude mice (at 6‐8 weeks of age) were purchased from Beijing HFK Bio‐technology. Mice were housed in a controlled environment (humidity, 50%‐60%). A total of five mice were housed per cage and were maintained at room temperature under a 12‐h light/dark cycle; Mice were provided free access to food and water.

### 2.3 Cell culture and treatment

The human cancer cell line A549 (BeNa culture collection, cat #BNCC337696), Human bronchial epithelial cell line (HBE, BeNa culture collection, catalog #BNCC338600) were cultured in Dulbecco's modified eagle medium (DMEM, HyClone) supplemented with 5% foetal bovine serum (FBS, Every Green, catalog #11011‐8611) and 1% penicillin/streptomycin, solution at 37°C in a humidified atmosphere containing 5% CO_2_. The medium was replaced every 3 days, and cells were subcultured or subjected to experimental procedures at 80%‐90% confluence.

### 2.4 Preparations of lentivirus and plasmids

Recombinant lentivirus (AMPK‐LV; siAMPK‐LV; siHK2‐LV; siHDAC4‐LV; siHDAC5‐LV; Vector‐LV) was prepared by GeneChem Company. Plasmids and the RNAi vector were successfully constructed and then packaged in 293T cells. The concentrated titre of virus suspension was 4 × 10^12^ Tu/L.

### 2.5 Establishment of stable cell lines

0.8 × 10^6^ A549 cells were seeded into 60‐mm plastic dishes. After the cells reached about 30%‐35% confluence, lentiviruses containing AMPK siRNA (sgAMPK) or HK2 siRNA were infected following the manufacturer's protocol at the desired multiplicity of infection (MOI = 10). After 8 hours, infection medium was removed and fresh medium was added. After an additional 64 hours, GFP co‐expression on the construct was used to determine efficiency of viral transduction.

### 2.6 Cellular proliferation, invasion and migration assays

In brief, CCK‐8 assay was applied to measure the cell proliferation according to the Cell Counting Kit‐8 manufacturer's protocol (Meilunbio, catalog #MA0218). For migration and invasion assays, the transwell filter inserts with a pore size of 8 μm were coated without or with matrigel (1:10 dilution), respectively. 5 × 10^4^ cells (for migration) and 1 × 10^5^ cells (for invasion) were, respectively, seeded in serum‐free medium in the upper chamber. After 24 h incubation at 37°C, cells in the upper chamber were carefully removed with a cotton swab and the cells that had traversed the membrane were fixed in methanol, stained with Crystal violet (0.04% in water; 100 μL) and counted the permeating cells under the inverted microscope and photographed.

### 2.7 Apoptosis

Apoptosis assay was carried out using the Annexin VFITC/PI apoptosis detection kit (KeyGen Biotech) according to the manufacturer's protocol. Briefly, 0.5 mL binding buffer was added into 1 × 10^5^ A549 and HBE cells. Sequentially, the cells were stained with PI at room temperature for 15 min and then analysed by flow cytometry (BD, FACSCantoTM II).

### 2.8 Immunofluorescence

HBE cells were fixed with 4% paraformaldehyde for 15 min, permeabilized with Triton X‐100 (0.1%) and blocked with solution containing 5% bovine serum before applying the primary antibody. Specimens were incubated, respectively, with anti‐E‐cadherin (Abcam, catalog #ab76055; 1:200) and anti‐N‐cadherin (Abcam, catalog #76057; 1:200), for 12 hours in 4°C. The specimens were subsequently incubated with secondary antibodies, Alexa Fluor 594 donkey anti‐rabbit (Life Technologies, catalog #R37119; 1:200) and fluorescein (FITC)‐conjugated AffiniPure Goat anti‐mouse IgG (Jackson ImmunoResearch, catalog #115‐095‐003) under light‐protected conditions for one hour at room temperature. Nuclei were stained with DAPI (4′, 6‐diamidino‐2‐phenylindole, KeyGen Biotech, catalog #KGA215‐10). After final washing, the coverslips were mounted on slides using 50% glycerin. Then, the sections were observed under a fluorescence microscope (Olympus).

### 2.9 Western blotting analysis

A list of the catalogue and batch numbers of commercially procured reagents is provided in Table S1. For the whole cell lysate, cells were lysed with a Cell Total Protein Extraction Kit (Sangon Biotech, catalog #C510003) containing a cocktail of phosphatase inhibitors and protease inhibitors. Primary antibodies for AMPK (Proteintech, catalog #10929‐2‐AP), p‐AMPK (Cell signaling, catalog #8208), Fibronectin (BD Transduction Laboratories, catalog #610077), Vimentin (Proteintech, catalog #10366‐1‐AP), N‐cadherin (Abcam, catalog #ab18203), E‐cadherin (Abcam, catalog #ab133597), Snail (Proteintech, catalog #13099‐1‐AP), HK1 (ABclonal, catalog #A1054), HK2 (ABclonal, catalog #A0094), LDHA (ABclonal, catalog #A1146), MCT‐1 (Proteintech, catalog #20139‐1‐AP), G6PI (Proteintech, catalog #15171‐1‐AP), TPI (ABclonal, A2579), HDAC1 (Proteintech, catalog #10197‐1‐AP), HDAC4 (Proteintech, catalog #17449‐1‐AP), HDAC5 (Proteintech, catalog #16166‐1‐AP) and β‐actin (Bioworld, catalog #AP0060) were followed by fluorescently labelled anti‐mouse (Biodragon Immunotech, catalog #BF03001) or anti‐rabbit antibodies (Biodragon Immunotech, catalog #03008), and the blot was then imaged using the Odyssey infrared imaging system (Li‐Cor). Western blots were quantified using ImageJ software. Protein levels were calculated from the ratio of corresponding protein/β‐actin.

### 2.10 RNA extraction and RT‐PCR analysis

TRIzol reagent was used to extract RNA from A549 and HBE cells. The cDNA was generated with random primers using the Reverse Transcription System (Promega). GAPDH was used for normalization of qRT‐PCR data. Primer sequences used in this study were listed in Table S2 ***.***

### 2.11 Extracellular flux measurements

Metabolic analyses were done with the Seahorse XFe96 Analyzer (Seahorse Bioscience), which measures the extracellular acidification rate (ECAR) and oxygen consumption rate (OCR) of live cells. Glycolytic rates were measured with the Seahorse XF glycolytic rate assay (S7805A, Seahorse Agilent).

### 2.12 High performance liquid chromatography

Cells were quickly extracted with pre‐cooled 80% methanol and assayed using a high performance liquid chromatography (HPLC) system. ATP levels were calculated by dividing the peak area of samples by standards.

### 2.13 Tumour xenograft study

5 × 10^6^ A549 cells with siAMPK treatment and control cells were injected subcutaneously into the flanks of mice to establish the animal models. Three weeks later, a portion of the mice were killed and the tumours were weighted. The other mice continued feeding. The huge tumours were harvested after 10 weeks. Thirty‐six mice were randomly divided into three groups as follows: (a) Control group (two mice died of tumour body rupturing); (b) Vector group (two mice died of tumour body rupturing); and (c) siAMPK group (two mice died of tumour body rupturing and two mice died of unknown causes). After opening the chest cavity, excise the internal organs and observe the medial chest wall of the mice. The incidence of intrathoracic metastasis was assessed by visualizing the distribution of tumours on the chest wall.

### 2.14 Statistical analysis

Numerical data are expressed as the mean ± SEM. Two independent sample data sets were tested using two‐tailed Student’s *t* test. Multiple group comparisons were evaluated by one‐way ANOVA followed by least significant difference *t* test for post hoc analysis. Chi‐square or Fisher's exact tests were used to compare categorical variables. Analyses were performed using SPSS software (SPSS, Inc.). *P* < .05 was considered as significant difference.

## 3 RESULTS

### 3.1 Low AMPK expression correlates with clinicopathologic parameters of NSCLC

**FIGURE 1:**
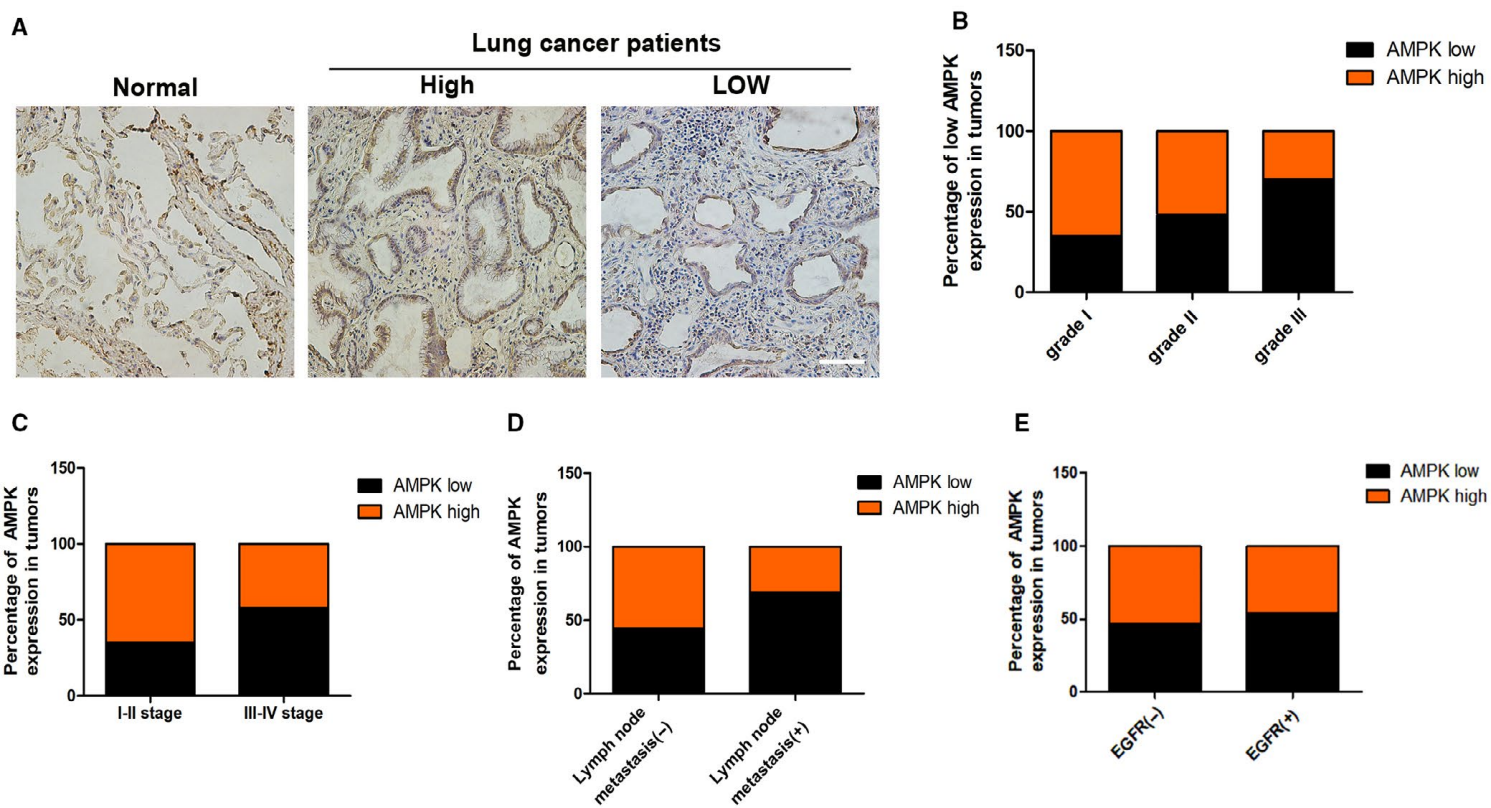
Low AMPK expression correlates with clinicopathologic parameters of NSCLCs. A, AMPK immunostaining in TMAs are shown, bar = 100 μm. B, Percentages of human lung cancer samples with low level of AMPK expression in different tumour grades. C, Correlation of AMPK expression with TNM stage. D, Correlation of AMPK expression with lymph node metastasis number. E, Correlation of AMPK expression with EGFR mutation. NSCLC, non–small‐cell lung cancer; TMA, tissues microarray

To investigate the expression of AMPK in lung cancer, we constructed tissue microarray (TMA) of 192 human NSCLC specimens, followed by immunohistochemical (IHC) analysis (Figure 1A). The correlation between AMPK level and the clinicopathologic characteristics were analysed (Table S3). Compared with histology grade 1, AMPK level in histology grade 3 was significantly lower (*P* = .022, *χ* ^2^ test; Figure 1B). The results also indicated that the low expression of AMPK was positively correlated with lymph node metastasis (*P* = .016, *χ* ^2^ test, Figure 1D) and tumour T stage (*P* = .026, *χ* ^2^ test, Figure 1C), but not with epidermal growth factor receptor (EGFR) mutation rate (*P* > .05, *χ* ^2^ test, Figure 1E).

### 3.2 AMPK is associated with proliferation and metastasis of lung cancer

**FIGURE 2:**
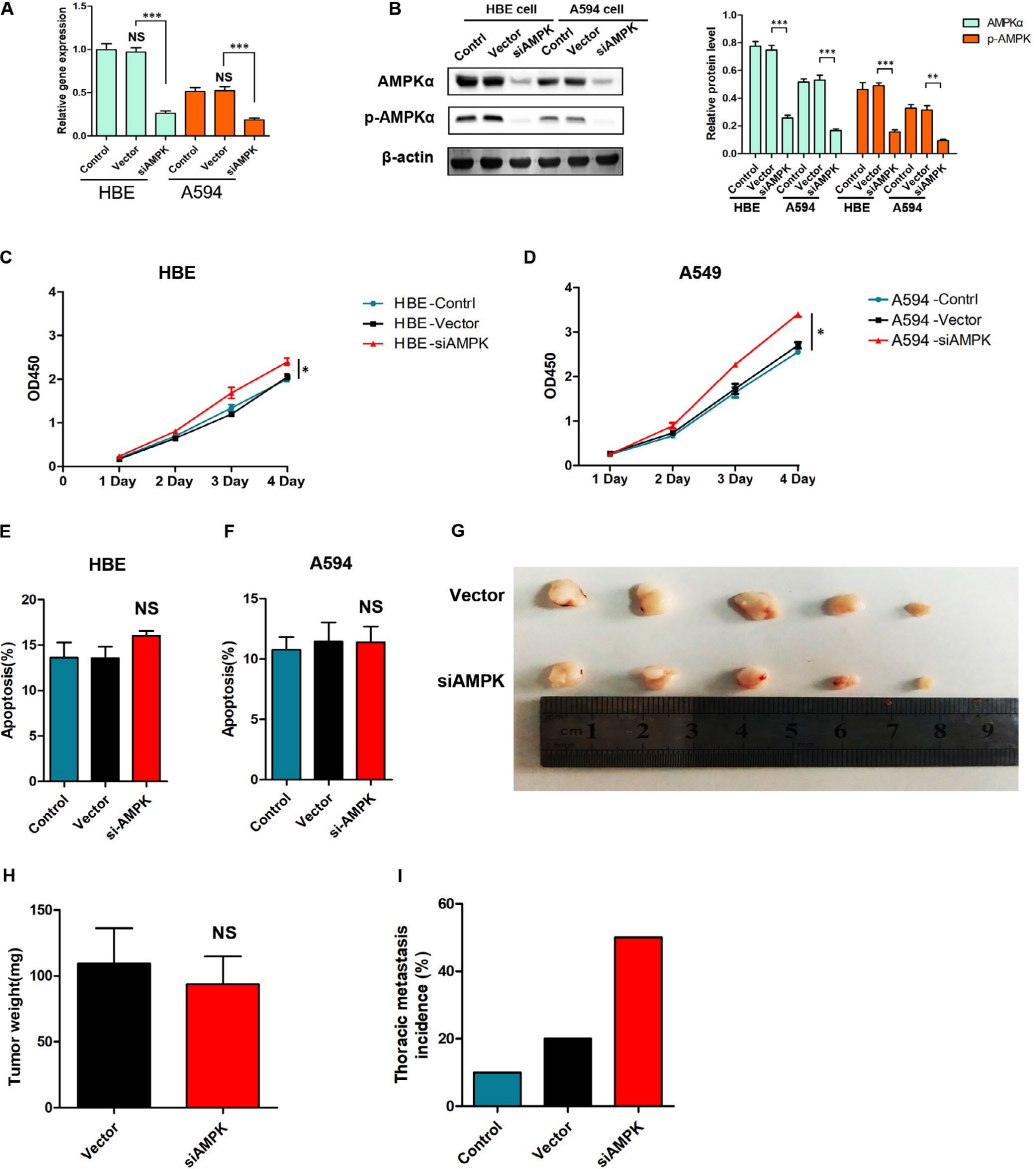
AMPK is associated with the proliferation and metastasis of lung cancer. A, RT‐PCR analysis of AMPK at mRNA level in HBE cells and A549 cells, *P* < .001 vs the indicated group, n = 3. B, Western blot determination of AMPK and p‐AMPK protein expression, *P < .05, **P < .01, ***P < .001 vs the indicated group, n = 3. C, D, CCK8 assay analysis of cell proliferation in HBE cells and A549 cells, *P < .05 vs the vector group, n = 3. E, F, Flow cytometric analysis of cells apoptosis in HBE cells and A549 cells, NS, *P* > .05 vs the vector group, n = 3. G, A549 cells were subcutaneously injected into BALB/c female nude mice, the lungs were harvested after 10 wk, and the intrathoracic metastasis of the tumour was observed. H, I, Photographs of matrigel plugs excised from mice after 3 wk of growth in vivo and quantitative analysis of the tumour weight, NS, *P* > .05 vs the vector group, n = 5

Human bronchial epithelial (HBE) and adenocarcinomic human alveolar basal epithelial (A549) cells were transiently transfected with siRNA directed against AMPK‐lentivirus (siAMPK‐LV) or vector‐LV. Next, the expression of AMPKα and phospho‐AMPKα (p‐AMPKα, Thr^172^) in HBE and A549 cells, and the knockdown efficiency of the virus, were examined by Western blot and quantitative real‐time PCR analysis (Figure 2A,B). We applied the cell counting kit‐8 (CCK‐8) assay to measure cells proliferation. Results showed that low AMPK expression slightly promoted the proliferation activity of A549 and HBE cells (Figure 2C,D). In addition, siAMPK‐LV treatment had no effect on apoptosis in A549 and HBE cells, as was shown by flow cytometric analysis (Figure 2E,F) (*P* > .05). We further constructed mice xenograft models by subcutaneous injection of treated A549 cells, to verify the effect of siAMPK‐LV on the proliferation of lung cancer cells (Figure 2G). There was no significant difference in tumour quality between the siAMPK‐LV treatment group and the empty vehicle control group (93.6 ± 21.1 g vs 109.4 ± 26.8 g [Vector], *P* > .05) (Figure 2H). Interestingly, the models harvested after 10 weeks showed significantly higher probability of chest wall metastasis in the siAMPK‐LV‐treated group (*P* = .02, *χ* ^2^ test), suggesting that low AMPK expression might be promoting lung cancer metastasis (Figure 2I).

### 3.3 Low AMPK induces EMT in HBE cells

**FIGURE 3:**
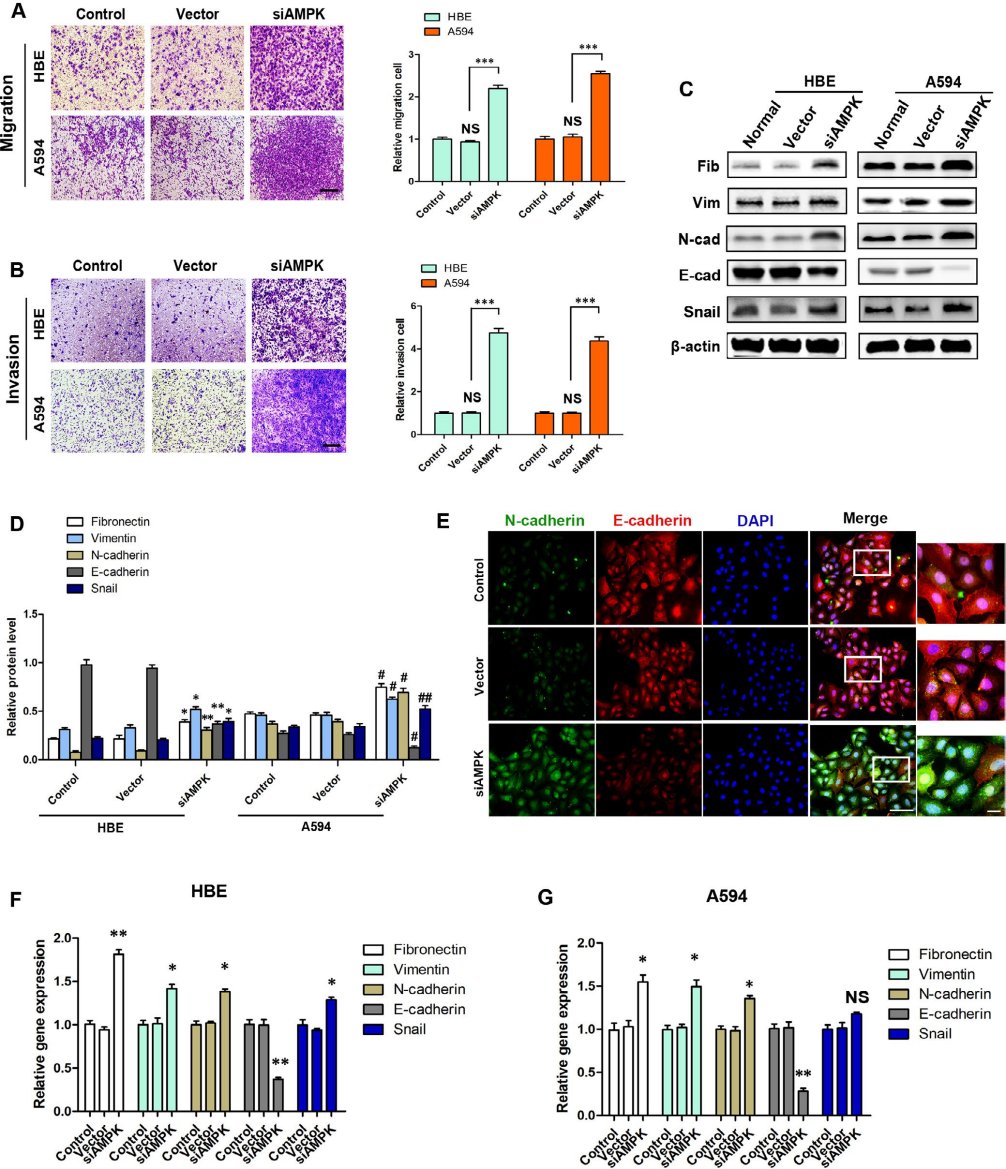
Low AMPK induces EMT in HBE cells. A, B, The migration and invasion of HBE cells and A549 cells with siAMPK and vector control, ***P < .001, NS, *P* > .05, vs the indicated group, bar = 100 μm, n = 3. C, D, Western blot determination of EMT‐related markers E‐cadherin, N‐cadherin, vimentin, fibronectin and snail protein expression, *P < .05, **P < .01 vs the vector group in HBE cells; #*P* < .05, ##*P* < .01 vs the vector group in A549 cells, n = 3. E, Immunofluorescence analysis of the expression of E‐cadherin (red) and N‐cadherin (green) in HBE cells, bar (left) = 10 μm, bar (right) = 5 μm. F, G, Relative mRNA expression levels of EMT markers and inducer in HBE cells and A549 cells, *P < .05, **P < .01 vs the respective vector group, n = 3

Our clinical data and result of the tumour xenograft study suggest that low AMPK expression is associated with lung cancer metastasis. We subsequently performed transwell assay to determine the effect of AMPK on A549 and HBE cells’ migration and invasion. Overexpression and knockdown treatments of AMPK were then applied to A549 cells. The results showed that siAMPK‐LV treatment significantly increased migration and invasion of HBE (Migration: 2.20 ± 0.08 vs 0.94 ± 0.03 [vector], *P* < .001; Invasion: 4.74 ± 0.21 vs 1.05 ± 0.09 [vector], *P* < .001) and A549 (Migration: 2.55 ± 0.06 vs 1.05 ± 0.07 [vector], *P* < .001; Invasion: 4.37 ± 0.19 vs 0.95 ± 0.08 [vector], *P* < .001) cells (Figure 3A,B). Western blot analysis showed that treatment of HBE cells with siAMPK‐LV induced down‐regulation of the tested epithelial marker (E‐cadherin), and up‐regulation of mesenchymal markers (vimentin, fibronectin and N‐cadherin) and EMT‐related transcription factor (snail), at the protein level (Figure 3C,D). Immunofluorescence assessment of HBE cells indicated that low expression of AMPK induced up‐regulation of N‐cadherin and down‐regulation of E‐cadherin (Figure 3E). PCR analysis revealed similar levels of mRNA expression, suggesting predominantly a transcription effect (Figure 3F,G). It is worth noting that reducing the expression of AMPK in tumour cells further promoted the loss of their original features and enhanced their ability to metastasize.

In addition, overexpression of AMPK effectively inhibited migration (0.48 ± 0.06 vs 0.94 ± 0.04 [vector], *P* < .01), invasion (0.39 ± 0.04 vs 0.96 ± 0.03 [vector], *P* < .001) and expression of mesenchymal markers in A549 cells (Figure S1). However, in HBEs cells, overexpression of AMPK had no effect on proliferation activity and apoptosis (Figure S2).

### 3.4 Low AMPK induces cell EMT by promoting glycolysis in HBE cells

**FIGURE 4:**
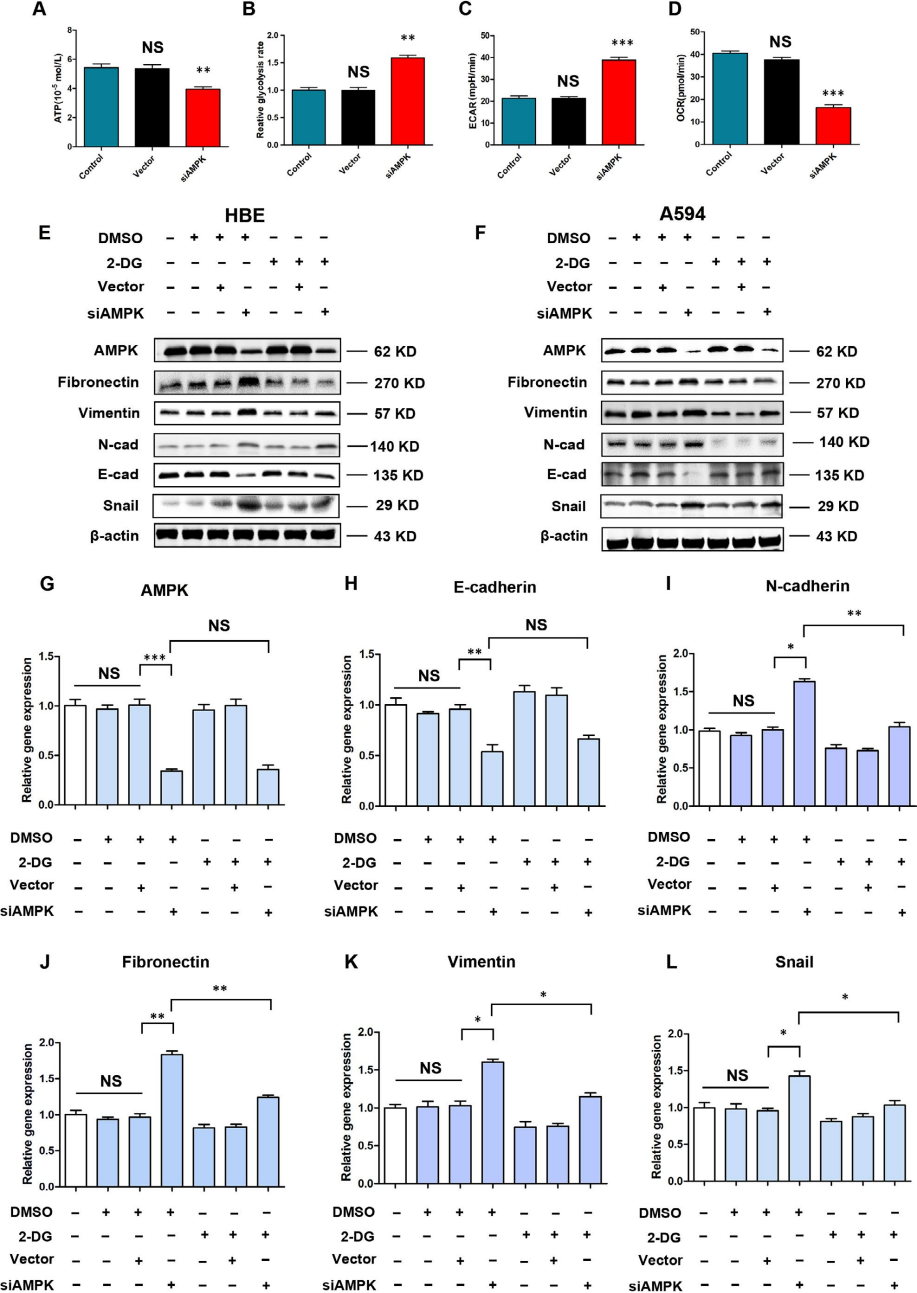
Low AMPK induces cell EMT by promoting glycolysis in HBE cells. A, High‐performance liquid chromatography (HPLC) analysis for ATP in HBE cells, **P < .01 vs the vector group; NS, *P* > .05 vs the control group, n = 3. B, Relative glycolysis rates in HBE cells, as judged by Seahorse analyses, **P < .01 vs the vector group; NS, *P* > .05 vs the control group, n = 3. C, D, Metabolic analyses were done with the Seahorse XFe96 Analyzer, which measures the ECAR and OCR of live HBE cells, ***P < .001 vs the vector group; NS, *P* > .05 vs the control group, n = 3. E, F, Western blot determination of EMT‐related markers in response to 2‐DG, n = 3. G‐L, Relative mRNA expression levels of EMT markers and inducer in HBE cells, *P < .05, **P < .01, ***P < .001, NS, *P* > .05 vs the indicated group, n = 3

Considering that AMPK is a key factor, regulating cell energy balance, we tried to determine the relationship between siAMPK‐LV and ATP production in HBE cells. We found that treatment of HBE cells with siAMPK‐LV decreased the OCR (16.38 ± 1.23 vs 37.58 ± 1.96 [Vector], *P* < 0.001), promoted glycolysis (1.59 ± 0.05 vs 0.95 ± 0.09 [Vector], *P* < .01) and ECAR (38.81 ± 2.21 vs 21.35 ± 0.85 [Vector], *P* < .001), and overall manifested mild inhibition on APT production (3.94 ± 0.16 vs 5.43 ± 0.26 [Vector], *P* < .01) (Figure 4A‐D and Figure S3). Clearly, low AMPK expression caused significant changes in metabolic characteristics in HBE cells. Interestingly, this process could be blocked by 2‐Deoxy‐D‐glucose (2‐DG), a competitive inhibitor of glycolysis (Figure S4). Based on these data, we suggested that EMT of HBE cells caused by siAMPK‐LV treatment is associated with an increase in glycolysis. Western blot analysis showed that 2‐DG blocked the down‐regulation of epithelial marker with high‐efficiency (E‐cadherin) and inhibited up‐regulation of interstitial markers (vimentin, fibronectin and N‐cadherin) caused by siAMPK‐LV treatment in both HBE and A549 cells (Figure 4E,F and Figure S5). The corresponding results of PCR analysis supported this conclusion (Figure 4G‐L).

### 3.5 Low AMPK promotes glycolysis by up‐regulating the expression of hexokinase 2 in HBE cells

**FIGURE 5:**
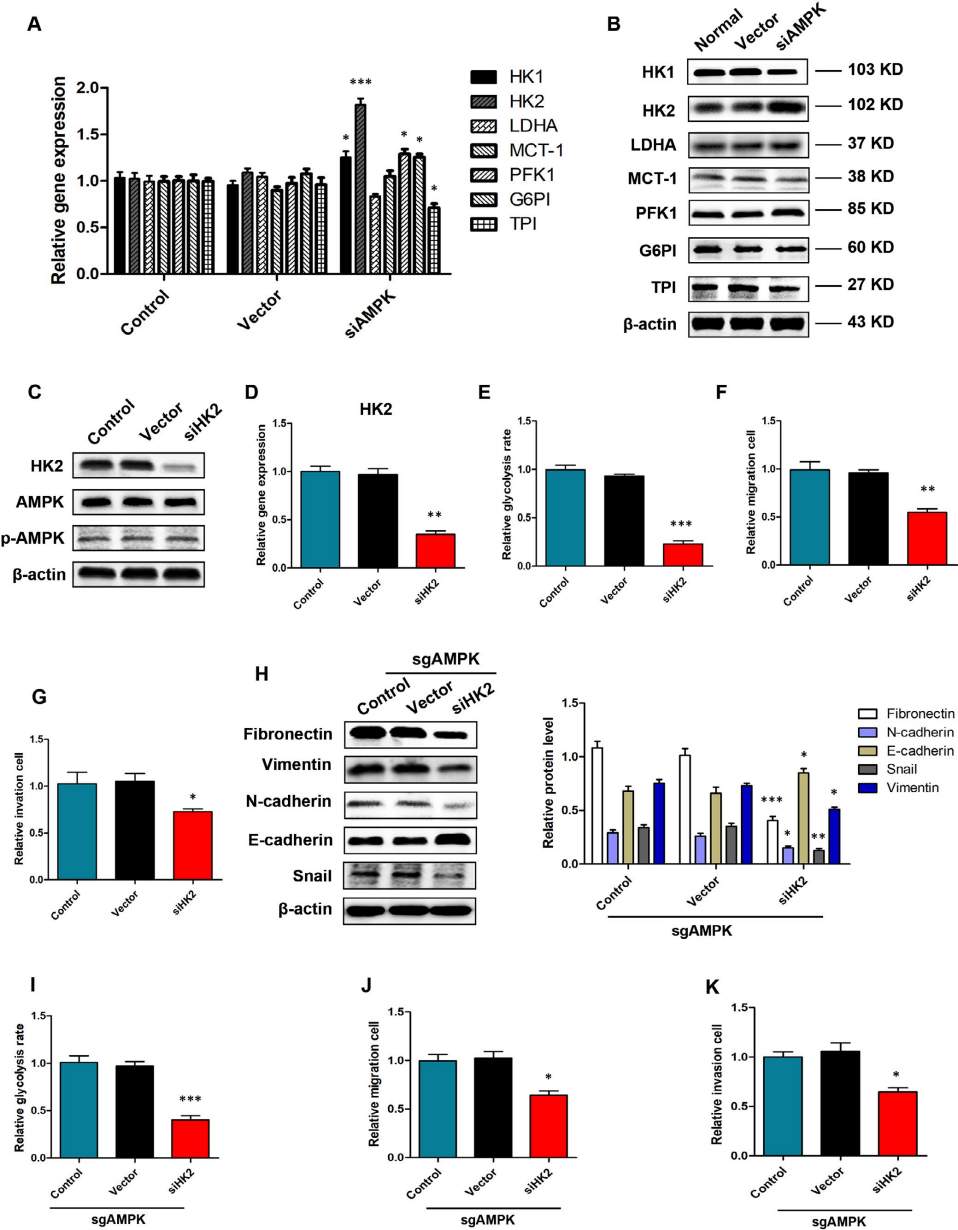
Low AMPK promotes glycolysis by up‐regulating the expression of HK2 in HBE cells. A, B, Relative mRNA and protein expression levels of HK1, HK2, LDHA, G6PI,TPI, MCT‐1 and PFK‐1 in HBE cells, *P < .05, ***P < .001, vs the Vector group, n = 3. C, D, Relative mRNA and protein expression levels of HK2, AMPK and p‐AMPK in HBE cells. E‐G, Relative glycolysis rates, migration, invasion in response to siHK2 treatment, *P < .05, **P < .01, ***P < .001 vs the respective vector group in HBE cells, n = 3. H, Western blot determination of EMT‐related markers protein expression in response to siHK2 treatment in sgAMPK HBE cells, *P < .05, **P < .01, ***P < .001 vs the vector group, n = 3. I‐K, Relative glycolysis rates, migration, invasion in response to siHK2 treatment, *P < .05, **P < .01, ***P < .001 vs the respective vector group in sgAMPK HBE cells, n = 3

The process by which cells acquire ATP and other intermediates through glycolysis requires the involvement of multiple metabolic enzymes. We further assessed the expression of various glycolysis‐related enzymes in HBE cells, such as hexokinases (HK1, HK2), lactate dehydrogenase A (LDHA), glucose phosphate isomerase (GPI) and more. The results showed that siAMPK‐LV treatment caused changes in the expression of various metabolic enzymes at the gene and protein levels, especially that of HK2 (Figure 5A,B). However, siHK2 treatment did not affect the expression levels of AMPK and p‐AMPK in HBE cells (Figure 5C,D). In addition, siHK2‐LV naturally caused a dramatic decrease of glycolysis rate (0.23 ± 0.03 vs 0.96 ± 0.03 [Vector], *P* < .001) that was accompanied by a decrease in the migration (0.54 ± 0.04 vs 0.93 ± 0.02 [Vector], *P* < .01) and invasion (0.73 ± 0.03 vs 1.05 ± 0.08 [Vector], *P* < .05) ability of HBE cells (Figure 5E‐G). Western blot analysis indicated that siHK2 significantly reduced the sgAMPK treatment‐induced high expression of interstitial markers, such as fibronectin, vimentin and N‐cadherin (Figure 5H). We also found that siHK2 prevented the increase in glycolysis rate (0.60 ± 0.04 vs 1.02 ± 0.07 [Vector], *P* < .001), secondary migration (0.64 ± 0.04 vs 1.06 ± 0.08 [Vector], *P* < .05) and invasion (0.64 ± 0.04 vs 1.06 ± 0.08 [Vector], *P* < .05) that were enhanced by sgAMPK treatment (Figure 5I‐K).

### 3.6 AMPK regulation of hexokinase 2 expression and of metabolism requires the involvement of histone deacetylases 4 and 5

**FIGURE 6:**
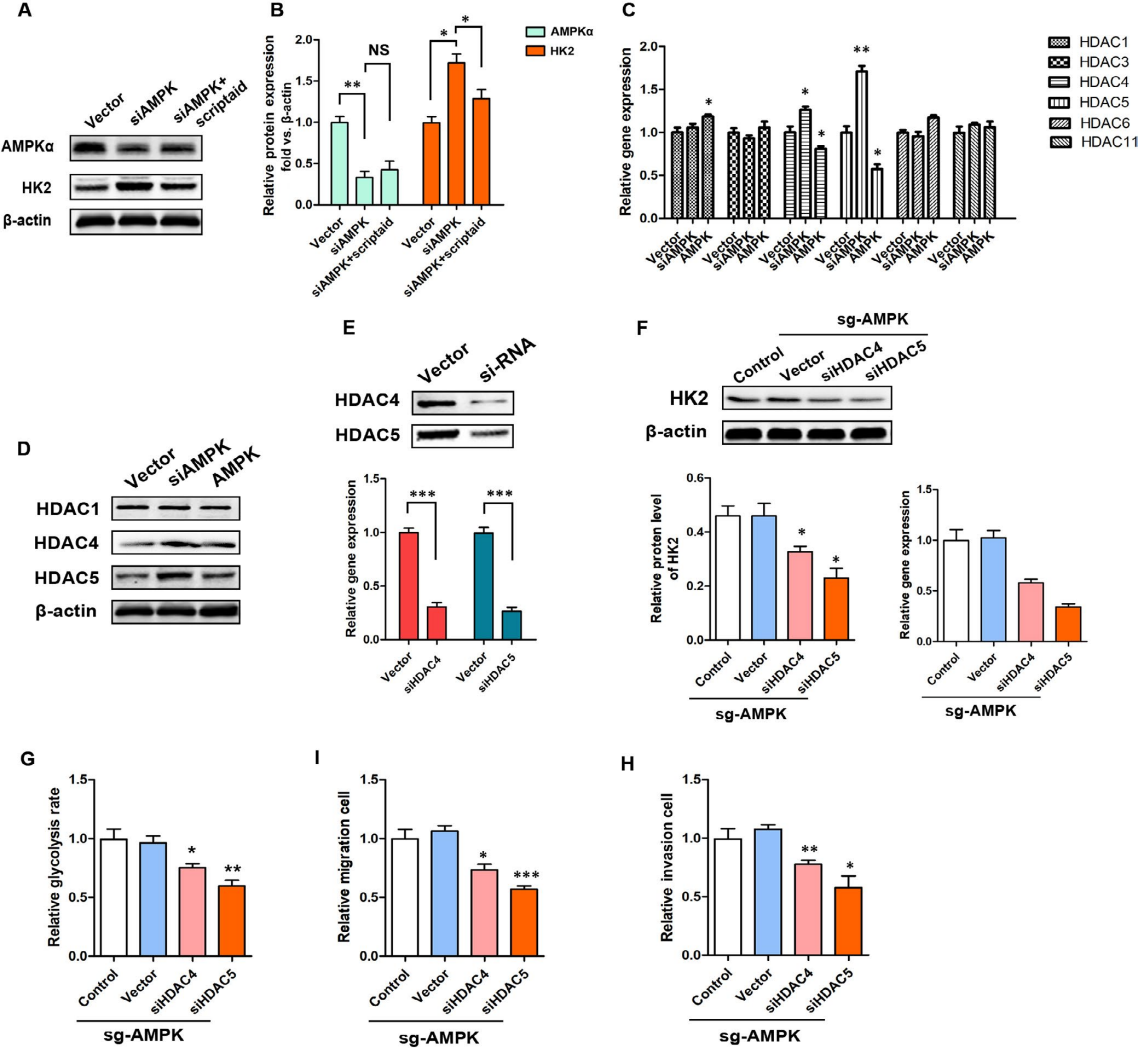
AMPK regulates the expression of HK2, and metabolism requires the involvement of HDAC4 and HDAC5. A, B, Relative mRNA and protein expression levels of AMPK and HK2 in response to scriptaid, *P < .05, **P < .01, NS, *P* > .05 vs the indicated group, n = 3. C, RT‐PCR analysis of HDACs at mRNA level in A549 cells with siAMPK and AMPK overexpression treatments, *P < .05, **P < .01 vs the vector group, n = 3. D, Western blot determination of HDAC1, HDAC4 and HDAC5 in A549 cells with siAMPK and AMPK overexpression treatments, n = 3. E, F, Relative mRNA and protein expression levels of HK2 in response to siHDAC4 and siHDAC5, *P < .05, **P < .01 vs the vector group, n = 3. G‐I, Relative glycolysis rates, migration, invasion in response to siHDAC4 and siHDAC5 in A549 cells with sgAMPK treatment, *P < .05, **P < .01, ***P < .001 vs the respective vector group, n = 3

The histone deacetylases (HDACs) are known to be involved in the transcription of various signals in the nucleus, including those related to metabolic pathways. To determine whether HK2 gene expression was regulated by HDACs, A549 cells were exposed to a single 60‐min treatment with HDACs inhibitor (Scriptaid, MedChemExpress, cat# HY‐15489). Western blot analysis showed that scriptaid partially counteracted siAMPK‐LV‐induced increase in HK2 expression at both protein (1.29 ± 0.09 vs 1.71 ± 0.10 [siAMPK‐LV], *P < *.05) and gene (1.24 ± 0.06 vs 1.64 ± 0.06 [siAMPK], *P *< .01) and had no effect on the expression of AMPK (*P* > .05) (Figure 6A,B and Figure S6) Therefore, we suggested that HDACs are involved in the mechanism by which AMPK regulates HK2 expression. Next, we examined the expression of HDAC1 (Class Ia), HDAC3 (Class Ib), HDAC4 (Class IIa), HDAC5 (Class IIa), HDAC6 (Class Ib) and HDAC11 (Class IV) in A549 cells, with high and low AMPK expression. The results suggest that HDAC4 and HDAC5 might play a mediating role in AMPK regulation of tumour cell metabolism (Figure 6C,D). We constructed HDAC4 and HDAC5 knockdown lentiviruses (Figure 6E) and subsequently transferred them into sgAMPK‐stable cell line. The results indicate that siHDAC4 and siHDAC5 significantly down‐regulated the expression of HK2 (0.30 ± 0.06 [siHDAC4] and 0.21 ± 0.04 [siHDAC5] vs 4.51 ± 0.13 [Vector], *P* < .05) in A549 sgAMPK cells (Figure 6F). From a functional point of view, siHDAC4 and siHDAC5 effectively reduced the high glycolysis level (0.75 ± 0.03 [siHDAC4] and 0.59 ± 0.04 [siHDAC5] vs 0.96 ± 0.06 [Vector], *P* < .05), migration (0.77 ± 0.03 [siHDAC4] and 0.57 ± 0.10 [siHDAC5] vs 1.07 ± 0.04 [Vector], *P* < .05) and invasive (0.73 ± 0.05 [siHDAC4] and 0.51 ± 0.03 [siHDAC5] vs 1.06 ± 0.04 [Vector], *P* < .05) abilities induced by high AMPK expression.

## 4 DISCUSSION

For patients with lung cancer, inhibiting the metastasis of tumour cells means extending their survival time and improving their quality of life. It is recognized that the metabolic characteristics of tumours present significant traits that promote proliferation, metastasis and immune evasion. The major finding in this study is that AMPK is an important regulator of lung cancer cells’ metabolism and is closely related to the histological grade and malignancy of these tumours.

In order to meet the needs of growth, survival and metastasis, cancer cells employ distinct metabolic pathway to acquire prodigious anabolic materials and products.^17^ Termed the Warburg effect, cancer cells deploy high glycolysis and glucose catabolism to produce ATP and lactic acid.^18^, ^19^ In addition, the formation of a tumour microenvironment that is resistant to the immune system defences, also depend on this particular metabolic approach.^20^, ^21^ Therefore, it is widely accepted that targeting tumour metabolism is a very promising treatment approach for cancer. Disturbingly, many metabolic pathways are shared between tumours and normal cells. Hence, disrupting the metabolism of tumour cells will inevitably be damaging normal cells. Such is the consequence of treatment with DON (6‐Diazo‐5‐oxo‐L‐nurleucine), a glutamine antagonist, which exhibits extraordinary anti‐tumour effects and unacceptable side effects.^22^

According to reports, AMPK serves as a metabolic tumour inhibitor, which can reduce the risk and/ or mortality of certain types of cancer, especially breast cancer, pancreatic cancer and prostate cancer. In the context of tumour development, cells of epithelial origin would transform into a mesenchymal‐like phenotype to obtain greater mobility, invasiveness and anti‐apoptosis. In this work, we present a comprehensive set of data, suggesting remarkable roles for AMPK in EMT, metabolic regulation and progression of lung cancer. Specifically, we first show that the regulator, AMPK, which is responsible for maintaining cellular energy homeostasis, has lower expression in A549 cells than in normal bronchial epithelial HBE cells. Second, siAMPK treatment up‐regulates the expression of various glycolysis‐limiting enzymes, including HK2, and subsequently increases the rate of glycolysis and lactic acid production in HBE cells. As expected, artificially up‐regulating the expression of AMPK significantly inhibited the level of glycolysis in A549 cells. It should be emphasized that knockdown of AMPK in A549 cells, further promoted the Warburg effect, allowing these tumour cells gain greater invasiveness. In contrast, overexpression of AMPK led to a reversal of EMT. In addition, it could be that the high level of AMPK in HBE cells, as a result of AMPK overexpression treatment, does not cause significant changes in AMPK at the protein level. This explains, to some extent, why overexpression of AMPK does not affect the proliferative activity and apoptosis of normal bronchial epithelial cells. Third, metabolic reprogramming is a critical step in meeting the demand of tumour cells proliferation, metastasis and immune evasion. Recent studies have shown that EMT can cause changes in a variety of metabolic enzyme activity and their expression levels, to reshape metabolic pathways.^23^ Metabolic reprogramming can also be fed back into the EMT mechanism, accompanied by the acquisition of enhanced migration and invasion ability.^24^ Here, low AMPK was shown to support proper function of the EMT transcription program, acting to down‐regulate epithelial markers and up‐regulate mesenchymal markers by regulating the expression of HK2 in A549 cells. Finally, clinical data show that AMPK expression levels are correlated with pathological parameters and progression of lung cancer. These data along with preclinical evidence supporting a novel therapeutic strategy to reverse mesenchymal phenotypes associated with invasion and metastasis.

Interestingly, according to existing reports, AMPK may have different or even opposite effects on metabolism or EMT progression in different cancer types. Chou et al^25^ proposed that AMPK can reverse the mesenchymal phenotype of cancer cells by targeting the Akt‐MDM2‐Foxo3a signaling axis, while Saxena et al^26^ hold that high level of AMPK was an accelerator of pathological EMT. This inconsistency may be related to the plasticity and background dependence of tumour characteristics. In this study, we found that AMPK is a negative regulator of the Warburg effect in NSCLC and that it suppresses EMT and tumour metastasis. This is in line with the findings of Jones et al^16^

It has been reported that HDACs are widely involved in nuclear transcriptional regulation of a variety of signals, mainly those involved in transcriptional repression. Research by Hargreaves et al^27^ shows that AMPK regulates GLUT4 transcription in skeletal muscle through HDAC5. There are also reports showing that AMPK‐HDAC5 pathway facilitates accumulation of HIF‐1a in the nucleus and functional activation of HIF‐1 by deacetylating Hsp70 in tumour cells.^28^ Here, HDAC4 and HDAC5 were shown to mediate the regulation of AMPK on tumour metabolism. Given the functional characteristics of HDACs, we believe that neither HDAC4 nor HDAC5 directly leads to an increase in HK2 transcription. We suggest that a key intermediate must exist between HDACs and HK2, mediating the regulation of HK2 expression levels and tumour metabolism by HDAC4 and HDAC5. It might be HIF, but more research is needed to support or refute this view.

To summarize, in this study we have demonstrated the remarkable efficacy of AMPK in regulating the metabolism and metastasis of lung cancer cells. At the mechanistic level, low AMPK up‐regulates HK2 expression and glycolysis levels through HDAC4 and HDAC5. Low AMPK‐induced metabolism can promote EMT progression in normal bronchial epithelial cells and lung cancer cells, which in turn increases the risk for tumour metastasis. Although more details are still need to elucidate the mechanism, our data uncovered the importance of AMPK in lung cancer progression.

## CONFLICT OF INTEREST

The authors report no relationships that could be construed as a conflict of interest.

## AUTHOR CONTRIBUTIONS

**Haitao Ma:** Conceptualization (lead); Funding acquisition (lead). **Shoujie Feng:** Data curation (lead); Project administration (lead); Writing‐original draft (lead). **Li Zhang:** Writing‐review & editing (lead). **Xiucheng Liu:** Formal analysis (equal). **Guangbin Li:** Writing‐original draft (supporting). **Biao Zhang:** Data curation (supporting). **Ziwen Wang:** Data curation (supporting). **Hao Zhang:** Funding acquisition (equal); Writing‐review & editing (equal).

## Supporting information

Supplementary Material

## Data Availability

The data sets used and/or analysed during the current study are available from the corresponding author on reasonable request.
