# Supplementary material for: Low levels of AMPK promote epithelial‐mesenchymal transition in lung cancer primarily through HDAC4‐ and HDAC5‐mediated metabolic reprogramming
Source: J Cell Mol Med. 2020 Jun 9;24(14):7789–801. doi: 10.1111/jcmm.15410 (PMC7348170; doi:10.1111/jcmm.15410)
Supplement: Supplementary file 1 — Supplementary Material [file JCMM-24-7789-s001.docx]

**
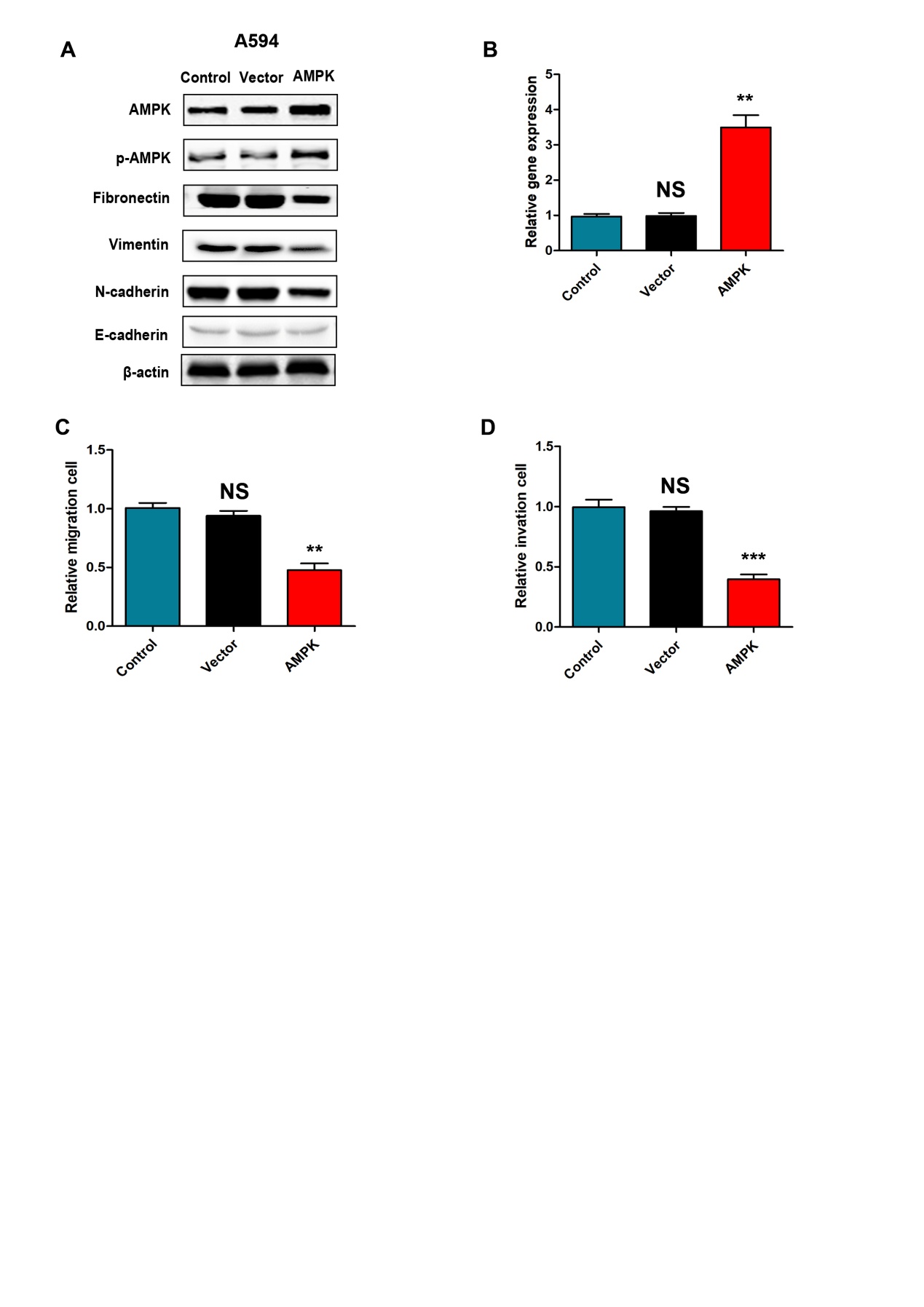
**

**Supplementary Figure 1.** The overexpression of AMPK effectively inhibits migration, invasion and the expression of EMT-related markers in A594 cells. (A) RT-PCR analysis for mRNA level of AMPK, n=3. (B) Western blot determination of EMT-related markers, n=3. (C, D) Relative migration, invasion in response to AMPK overexpression in A549 cells, n=3. **P＜0.01，***P＜0.001 vs the vector group; NS, P＞0.05 vs the control group.


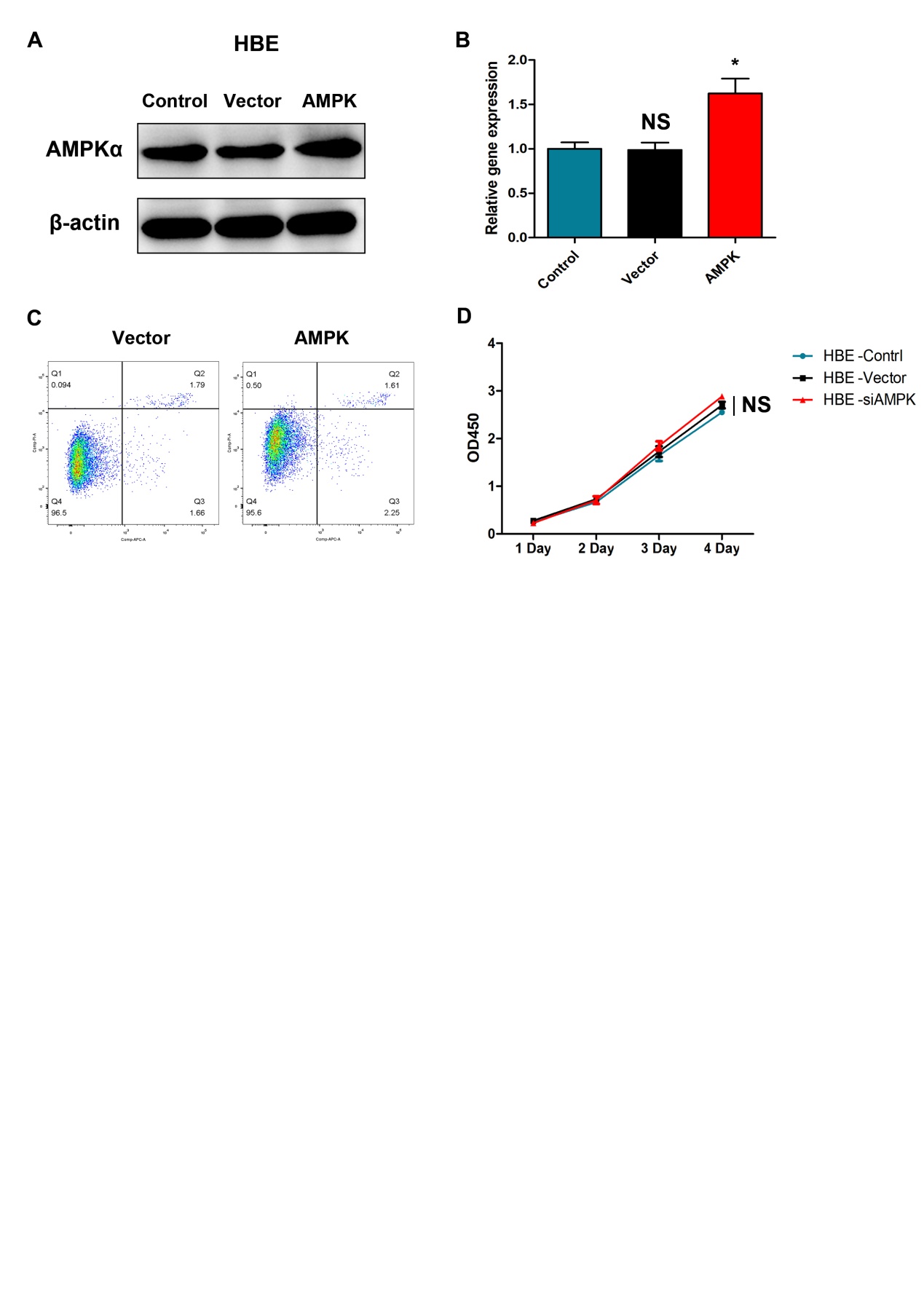


**Supplementary Figure 2.** The overexpression of AMPK have no effect on proliferation activity and apoptosis in HBEs cells. (A, B) Relatvie protein and mRNA level of AMPK in HBE cells, *P＜0.05 vs the vector group; NS, P＞0.05 vs the control group, n=3. (C) Flow cytometric analysis followed by Annexin PI staining, n=3. (D) CCK8 assay analysis of cell proliferation in HBE cells, NS, P＞0.05 vs the vector group, n=3.


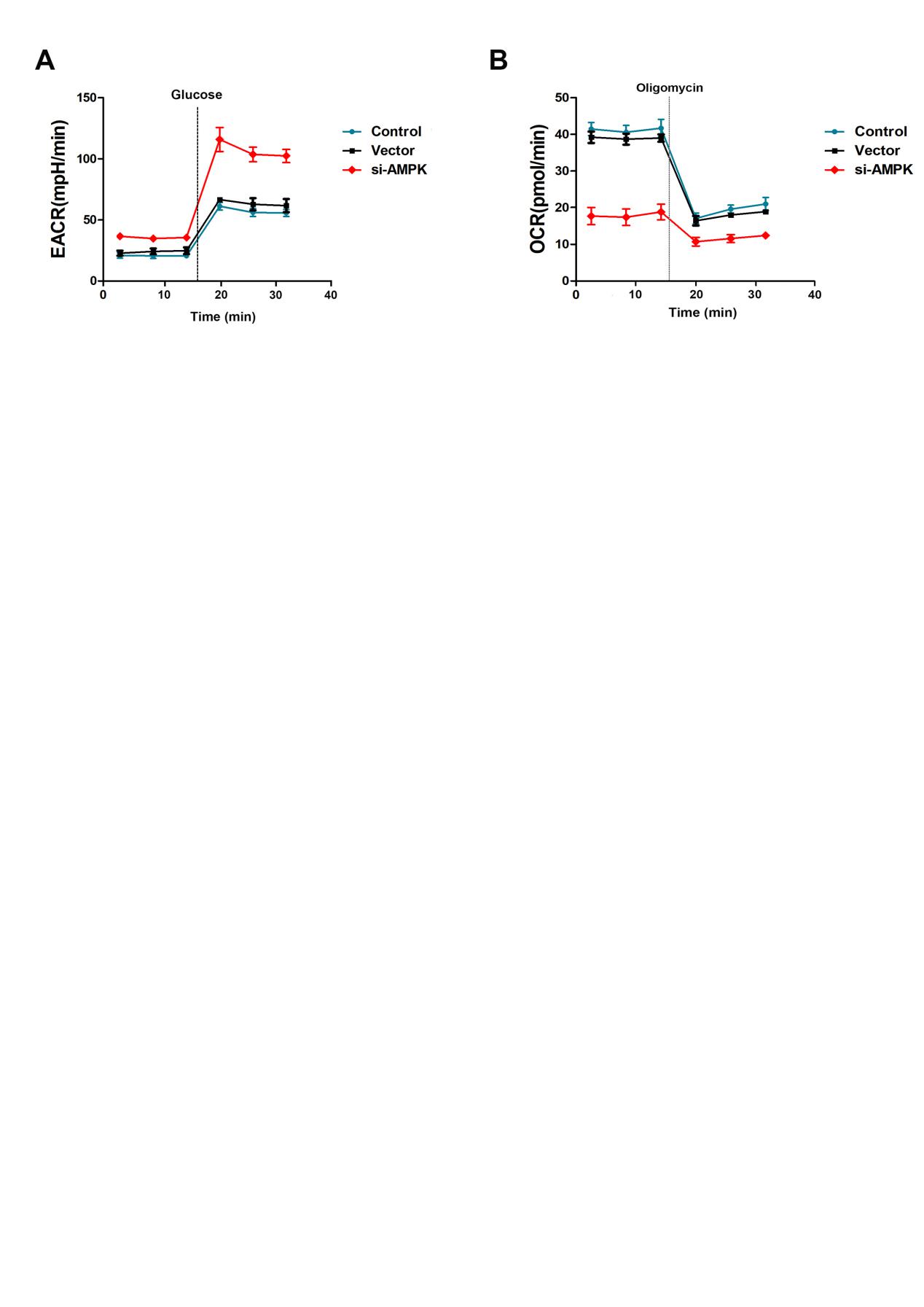


**Supplementary Figure 3.** The kinetic EACR and OCR plots of live HBE cells measured by Seahorse XFe96 Analyzer, n=3.


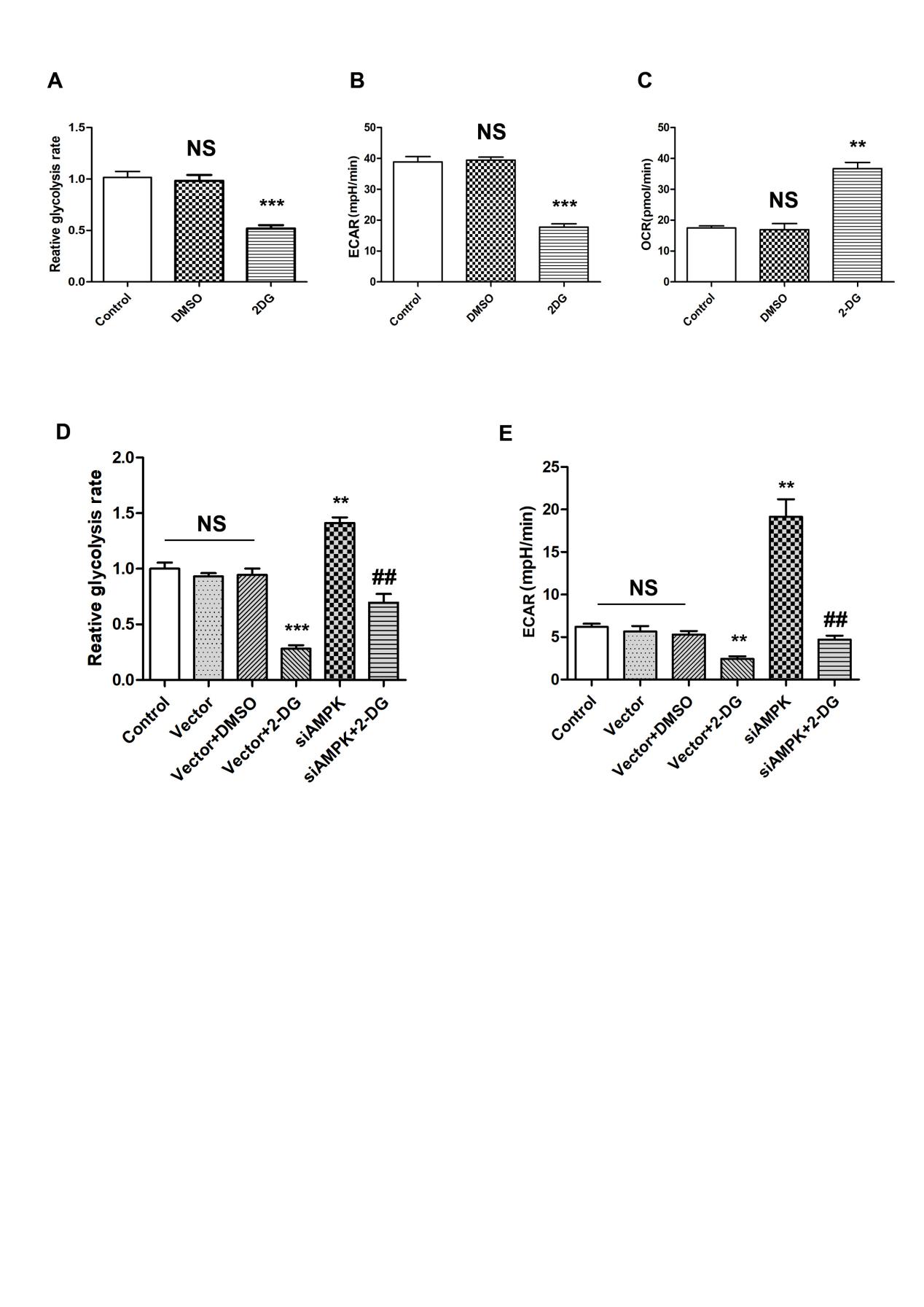


**Supplementary Figure 4.** 2-DG effective reducing of the Warburg effect caused by the siAMPK treatment in HBE cells. (A-C) Relative glycolysis rates, EACR and OCR in response to 2-DG in HBE cells, **P＜0.01，***P＜0.001 vs the vector group; NS, P＞0.05 vs the control group, n=3. (D, E) Relative glycolysis rates and EACR in response to 2-DG in HBE cells with siAMPK treatment, **P＜0.01，***P＜0.001 vs the vector group; NS, P＞0.05 vs the indicated group; ##P＜0.01 vs the siAMPK group, n=3.


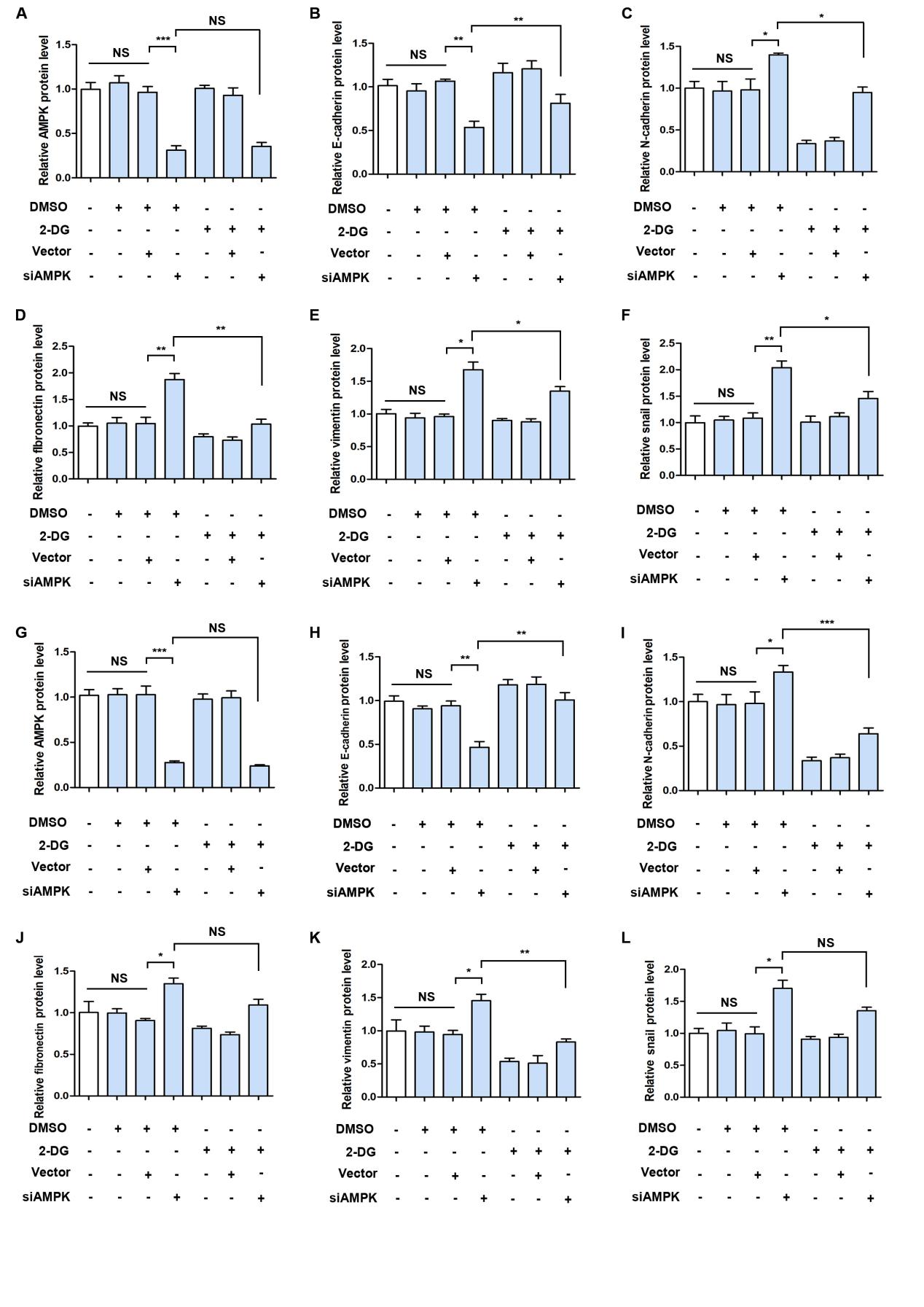


**Supplementary Figure 5.** Quantification of the protein levels of EMT-related markers in response to 2-DG in HBE cells. (A-F) Relative AMPK, E-cadherin, N-cadherin, fibronectin, vimentin and snail protein level in HBE cells. (G-L) Relative AMPK, E-cadherin, N-cadherin, fibronectin, vimentin and snail protein level in A549 cells. *P＜0.05, **P＜0.01, ***P＜0.001, NS，P＞0.05 vs the indicated group, n=3.


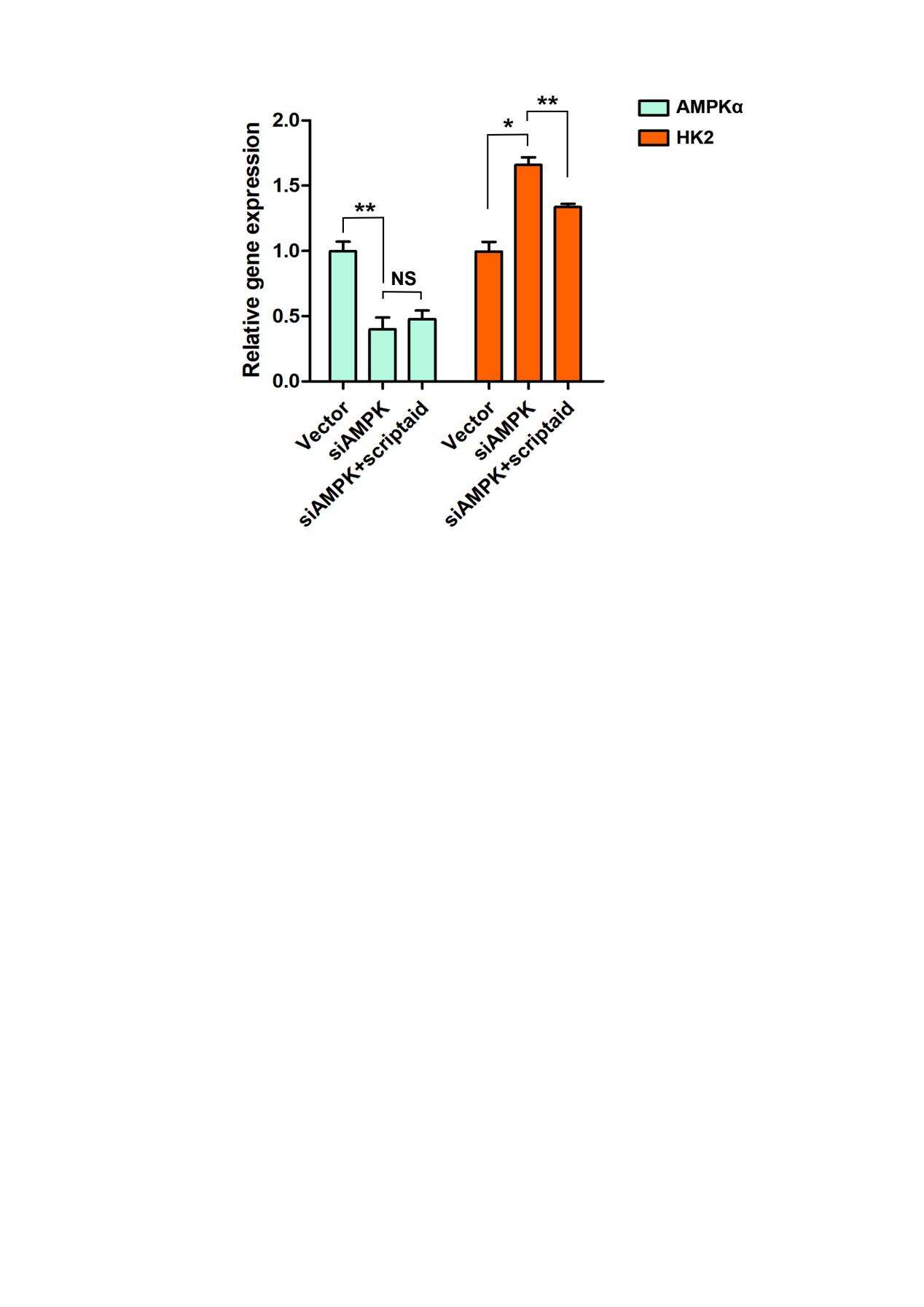


**Supplementary Figure 6.** Relative mRNA and protein expression levels of AMPK, and HK2 in response to scriptaid. *P＜0.05, **P＜0.01, NS, P＞0.05 vs. the indicated group, n=3.

**Supplementary Table 1: A list of the catalogue and batch numbers of commercially procured reagents**

|  | **Brand** | **catalog** | **lot** |
| --- | --- | --- | --- |
| Cell Total Protein Extraction Kit | Sangon Biotech | C510003 | -/- |
| Matrigel | BD Biocoat | 356234 | 9014341 |
| DAPI | KeyGen Biotech | KGA215–10 | 20190513 |
| FBS | Every GReen | 11011-8611 | 19090501 |
| Penicillin/streptomycin | VICMED | VC2003 | 0708A19 |
| Trypsin-EDTA Solution | Beyotime | C0201 | -/- |
| Cell Counting Kit-8 | Meilunbio | MA0218 | L-Aug-07E |
| DMEM | HyClone | SH30022.01 | AE29005269 |
| Annexin VFITC/PI apoptosis detection kit | KeyGen Biotech | KGA1030-50 | 20190124 |
| ***Antibodies used for WB:*** |  |  |  |
| Anti-N-cadherin | Abcam | ab18203 | GR122949-1 |
| Anti-E-cadherin | Abcam | ab133597 | GR97675-26 |
| Fibronectin | BD-Transduction Laboratories | 610077 | 4073761 |
| Vimentin | Proteintech | 10366-1-AP | 00067599 |
| Snail | Proteintech | 13099-1-AP | 00059200 |
| AMPK | Proteintech | 10929-2-AP | 00005201 |
| p-AMPK | Cell signaling | 8208 | -/- |
| HK1 | ABclonal | A1054 | 2758080 |
| HK2 | ABclonal | A0094 | 2757513 |
| TPI | ABclonal | A2579 | 2764465 |
| MCT1 | Proteintech | 20139-1-AP | 00061849 |
| LDHA | ABclonal | A1146 | 2758572 |
| HDAC 1 | Proteintech | 10197-1-AP | 00045620 |
| HDAC 4 | Proteintech | 17449-1-AP | 00018711 |
| HDAC 5 | Proteintech | 16166-1-AP | 00007602 |
| β-actin | Bioworld | AP0060 | AA24142 |
| Goat anti-Rabbit IgG | Biodragon Immunotech | BF03008 | KIA9044687 |
| Goat anti-Mouse IgG | Biodragon Immunotech | BF03001 | KIA9043917 |
| FITC-conjugated Affinipure Goat anti-Mouse IgG | Jackson Immuno-  Research | 115-095-003 | 129395 |
| Alexa Fluor TM 594 | Life Technologies | R37119 | 2072295 |

**Supplementary Table 2: Primer sequences used in this study**

| **Gene** | **Forward (5’-3’)** | **Reverse (5’-3’)** |
| --- | --- | --- |
| AMPK | GGCTGGAGTGCAATGGTGTGG | AGGAAGCTGAGGCGGGAGAATC |
| E-cadherin | GACAACAAGCCCGAATT | GGAAACTCTCTCGGTCCA |
| Fibronectin | TCCCTCGGAACATCAGAAAC | GAGAACTTTGCCGTTGAAGC |
| N-cadherin | CGGGTAATCCTCCCAAATCA | CTTTATCCCGGCGTTTCATC |
| Vimentin | GAGAACTTTGCCGTTGAAGC | GCTTCCTGTAGGTGGCAATC |
| Snail | GCAAATACTGCAACAAGG | GCACTGGTACTTCTTGACA |
| HK1 | GCAGGGACCACACTTTGAGAACC | AGAGGAGGAAGAGGACAGCACAC |
| HK2 | GTGAACGATGCTCCTGCTCTGAAG | CTCCTCAACGGCAGCCACAATG |
| LDHA | CCGCTCGAGCTATGGCAACTCTAAAGGATCAGC | CGGAATTCTTAAAATTGCAGCTCCTTTTGG |
| MCT-1 | ACGCCAACGTACATCCCGAT | CGTCAGAAC CCGACTCCTCC |
| G6PI | AACCGCTCCAACACTCCCATTAAC | TCGCCACTACGCACTTTATGACAG |
| TPI | AGTCTCGAGTCACTGCTAGCGTTAAC | ATCGGATCCCCCATGCGTGTAAATT |
| HDAC1 | ACGACGGGGATGTTGGAAAT | TGGCTTTGTGAGGGCGATAG |
| HDAC3 | GAGGGATGAACGGGTAGACA | CAGGTGTTAGGGAGCCAGAG |
| HDAC4 | GTGATGGGATTTCCATTGAT | CAGTGGTTCAGATTCCGGTGG |
| HDAC5 | GGAATTCATGAAGTTGGAGGTGTTCGTC | CCTCGAGCGCTACTCAGGCTAGG AGCGTCTCCAC |
| HDAC6 | GCTCTAGACATGACCTCAACCGGCCAGG | CCCAAGCTTTTAGTGTGGGTGGGGCATATC |
| HDAC11 | CGGGATCCATGClACACACAACCCAGCTGTACC | CGGAATTCTCAGGGCACTGCAGGGGGAA |
| β-actin | CATCCGTAAAGACCTCTATGCCAAC | ATGGAGCCACCGATCCACA |

**Supplementary Table 3: AMPK staining and clinicopathological characteristics of 192 lung cancer patients**

| **Variables** | **AMPK staining** | | | | |
| --- | --- | --- | --- | --- | --- |
|  |  | | | | |
|  | **Low (%)** | **High (%)** | **Total** | | ***P* *** |
| **Age** |  |  |  |  | |
| ≤50 | 22(43.1) | 29(56.9) | 51 | 0.633 | |
| >50 | 48(41.0) | 69(58.9) | 117 |  | |
| **Histology grade** |  |  |  |  | |
| Grade I | 25(40.9) | 30(59.0) | 61 | 0.022 | |
| Grade II | 26(49.1) | 27(50.9) | 53 |  | |
| Grade III | 30(76.9) | 9(23.1) | 39 |  | |
| **Lymph node metastasis** |  |  |  |  | |
| Negative | 24(48.0) | 26(52.0) | 50 | 0.016 | |
| Positive | 60(75.9) | 19(24.1) | 79 |  | |
| **Tumor stage** |  |  |  |  | |
| I~II | 26(40.6) | 38(59.4) | 64 | 0.026 | |
| III~IV | 47(58.2) | 34(41.9) | 81 |  | |
| **EGFR status** |  |  |  |  | |
| Negative | 19(46.3) | 22(53.7) | 41 | 0.711 | |
| Positive | 14(56.0) | 11(44.0) | 25 |  | |

^*^ *P* values are from χ^2^ test.

Some cases were not available for the information.
